# Impact of a specialised palliative care intervention in patients with advanced soft tissue sarcoma – a single-centre retrospective analysis

**DOI:** 10.1186/s12904-020-00702-1

**Published:** 2021-01-14

**Authors:** F. Brandes, J. K. Striefler, A. Dörr, M. Schmiester, S. Märdian, G. Koulaxouzidis, D. Kaul, A. Behzadi, P. Thuss-Patience, J. Ahn, U. Pelzer, L. Bullinger, A. Flörcken

**Affiliations:** 1 Department of Hematology, Oncology, and Tumor Immunology, Campus Virchow-Klinikum, Charité–Universitätsmedizin Berlin, corporate member of Freie Universität Berlin, Humboldt-Universität zu Berlin, and Berlin Institute of Health, Augustenburger Platz 1, 13353 Berlin, Germany; 2 Charité–Universitätsmedizin Berlin, corporate member of Freie Universität Berlin, Humboldt-Universität zu Berlin, and Berlin Institute of Health, Centre for Musculoskeletal Surgery, Campus Virchow-Klinikum, Berlin, Germany; 3 Department of Surgery, Campus Virchow-Klinikum, Charité–Universitätsmedizin Berlin, corporate member of Freie Universität Berlin, Humboldt-Universität zu Berlin, and Berlin Institute of Health, Plastic and Reconstructive Surgery, Berlin, Germany; 4 Department of Radiation Oncology, Campus Virchow-Klinikum, Charité–Universitätsmedizin Berlin, corporate member of Freie Universität Berlin, Humboldt-Universität zu Berlin, and Berlin Institute of Health, Berlin, Germany; 5 German Cancer Consortium (DKTK), partner site Berlin, and German Cancer Research Center (DKFZ), Heidelberg, Germany

**Keywords:** Palliative care, Soft tissue sarcoma, MIDOS symptom score, Symptom burden, Early palliative care intervention, Pain, Inpatient palliative care

## Abstract

**Background:**

Soft tissue sarcomas (STS) account for less than 1% of all malignancies. Approximately 50% of the patients develop metastases with limited survival in the course of their disease. For those patients, palliative treatment aiming at symptom relief and improvement of quality of life is most important. However, data on symptom burden and palliative intervention are limited in STS patients.

**Aim:**

Our study evaluates the effectiveness of a palliative care intervention on symptom relief and quality of life in STS patients.

**Design/setting:**

We retrospectively analysed 53 inpatient visits of 34 patients with advanced STS, admitted to our palliative care unit between 2012 and 2018.

Symptom burden was measured with a standardised base assessment questionnaire at admission and discharge.

**Results:**

Median disease duration before admission was 24 months, 85% of patients had metastases. The predominant indication for admission was pain, weakness and fatigue. Palliative care intervention led to a significant reduction of pain: median NRS for acute pain was reduced from 3 to 1 (*p* < 0.001), pain within the last 24 h from 5 to 2 (*p* < 0.001) and of the median MIDOS symptom score: 18 to 13 (*p* < 0.001). Also, the median stress level, according to the distress thermometer, was reduced significantly: 7.5 to 5 (*p* = 0.027).

**Conclusions:**

Our data underline that specialised palliative care intervention leads to significant symptom relief in patients with advanced STS. Further efforts should aim for an early integration of palliative care in these patients focusing primarily on the identification of subjects at high risk for severe symptomatic disease.

## Key statements

**What is already known about the topic?**

- In a variety of advanced cancer diseases integration of palliative care has a significant impact on patient outcome, quality of care, length of hospital stay and hospital costs and leads to a less aggressive therapeutic approach during end-of-life care as well as to more contentment in patients and their relatives
- The early integration of palliative care leads to an overall survival (OS) benefit
- Only limited data are available on the specific challenges of palliative care interventions in the context of soft tissue sarcoma (STS) and there are no published data concerning an early integration of palliative care in patients with sarcoma

**What this paper adds**

- This is the first report in STS patients analysing hospital-based palliative care intervention, which does not focus on end-of-life care, but on palliative care intervention throughout the entire course of the disease
- The interventions resulted in a significant reduction of pain, an improvement of symptom burden and a decreased stress level

**Implications for practice, theory or policy**

- Our analysis demonstrates that specialised palliative care intervention leads to a significant symptom relief throughout the entire course of the disease
- Further exploration of the effects of early integration of palliative care on symptom relief, quality of life and the possible improvement of overall survival in STS patients is warranted

## Background

Soft tissue sarcomas (STS) are rare and account for less than 1% of malignancies [1, 2]. Originating from mesenchymal tissue, they represent a heterogeneous group with more than 100 different histopathologically defined tumours.

Comprising all disease stages, the 5-year overall survival is 50 to 60%. Nevertheless, metastases occur in up to 50% of cases resulting in a poor outcome with overall 5-year survival of ~ 15%, only [3]. For patients with advanced disease, chemotherapy is the standard of care to prolong survival and improve the quality of life. In this setting, the median overall survival (OS) is 12.8 to 14.3 months, progression-free survival (PFS) is 4.6 to 7.4 months for doxorubicin monotherapy and doxorubicin combination therapy, respectively [4]. These data demonstrate a high unmet need for modern, effective therapies for this group of patients and highlight the importance of adequate palliative care strategies. Published data on the symptom prevalence and severity in advanced STS remain limited. Nevertheless, they sufficiently describe the high symptom burden and demonstrate the need for specialised diagnostics and optimised care [5–7]. Additionally, since OS remains reduced, the importance of palliative care needs to be increasingly spotlighted.

The WHO defines palliative care as follows: “an approach that improves the quality of life of patients and their families facing the problems associated with life-threatening illness, through the prevention and relief of suffering by means of early identification and impeccable assessment and treatment of pain and other problems, physical, psychosocial and spiritual “ [8]. Palliative care is also defined as „applicable early in the course of illness, in conjunction with other therapies that are intended to prolong life “ [8], and a relevant number of analyses have addressed the benefit of early integration of palliative care into cancer care [9]. According to Jack et al., cancer patients in general benefit from a hospital-based palliative care intervention regarding symptom relief, with the largest effects on pain and anorexia [10]. Data about palliative care intervention in the context of STS are sparse, and most authors concentrate on the description of symptom burden rather than assessing the symptom relief achieved through palliative care intervention. For example, Kawashima et al. reported that 93 and 78% of STS patients suffer from pain and nausea, respectively, and that a total of 98% of STS patients requires opioids within the last 2 weeks of life [11].

Our study aimed to assess the patterns of symptoms in STS patients (n_p_ = 34) who have been treated in a hospital-based palliative setting (n_i_ = 53 interventions), and we further intended to measure the benefit and effectiveness of a hospital-based palliative care intervention regarding symptom relief and quality of life in these patients.

In the context of STS, an optimal assessment for quality of life is not yet defined. During the last years, several tools, e.g. standardised questionnaires, electronic patient self-reporting and assessment by medical professionals, have been evaluated. For example, Gough et al. used questionnaires as well as semi-structured interviews in a mixed methods longitudinal study in patients with advanced STS [12]. In the analysis published by Schuler et al., patient-reported outcomes (PROs) were utilised to evaluate overall quality of life (QoL) in patients undergoing a palliative chemotherapy [13]. Hentschel et al. performed multiple standardised assessments for QoL in patients receiving systemic therapy and the value of additional expert-consented supportive treatment recommendations [14].

To our knowledge, there are only sparse data analysing hospital-based palliative care in STS patients primarily focusing on early interventions throughout the entire course of the disease, instead of end-of-life care.

## Methods

We conducted a retrospective analysis of patients with symptomatic advanced STS who were admitted to our palliative care unit at the Charité between 2012 and 2018. The analysis was performed after patients’ consent and according to the local ethical guidelines. Prerequisite for inclusion in the analysis was the availability of data both for admission and discharge.

This analysis included n_i_ = 53 interventions of n_p_ = 34 STS patients who presented with different STS subtypes (leiomyosarcoma as the most frequent subtype). Additionally, we gathered information on disease duration and prior therapies. Please see Table 1 for detailed patients’ characteristics.

**Table 1:** Patients’ characteristics

Characteristic	Total (***n*** = 34)
**Gender n (%)**	
male	19 (54)
female	15 (46)
**Age at admission (years)**	
mean (range)	59 (21–80)
**Tumour subtypes n (%)**	
Leiomyosarcoma	6 (17)
Angiosarcoma	4 (12)
Liposarcoma	3 (9)
MPNST	3 (9)
Pleomorphic sarcoma	3 (9)
Synovial sarcoma	3 (9)
Others	12 (35)
**Tumour stage at diagnosis n (%)**	
curative	14 (41)
locally adcanced	7 (21)
metastasized	13 (38)
**Tumour stage on admission n (%)**	
locally advanced	5 (15)
metastasized	29 (85)
**Disease duration until admission (months)**	
mean (range)	24 (1–125)
**Patients with initial surgery n (%)**	
yes	25 (74)
no	9 (26)
**Patients with previous treatment lines n (%)**	
0	2 (6)
≥ 1	32 (94)
**Patients with previous radiation n (%)**	
yes	25 (74)
no	9 (26)
**Palliative care interventions n (%)**	
1	22 (67)
2	4 (12)
3 or more	7 (21)

*n* number*, MPNST* malignant peripheral nerve sheath tumor

The palliative care unit at Charité Virchow-Klinikum belongs to the oncologic department and consists of an inpatient ward with ten single bed rooms. The patients are treated by a multidisciplinary team, including specialised palliative care physicians, palliative care nurses, physiotherapists, psychologists, dietists, musical therapists and social service. Patients are either admitted from home or transferred from other departments within the Charité/ external clinics. Patients known to our oncologic department, which was the case for all sarcoma patients, can be admitted/transferred without prior assessment of our palliative consulting service. For all other patients, the palliative consulting service supervises the indication for an admission to the palliative care unit. If no admission is realised, the team simply counsels and evaluates the needs of patients with palliative malignancies/ chronic diseases. The aim for all patients is an early integration of our palliative consulting service to accompany these patients throughout the course of their disease. For patients admitted to our ward, an individualised therapeutic plan based on the patient’s symptom burden is set up after the primary assessment, thoroughly documented and individually adjusted daily according to the standards of specialised inpatient palliative care intervention defined by the German Association for Palliative Medicine [15]. The therapeutic interdisciplinary interventional approach is a combination of pharmacological pain and symptom relief, conversational psychotherapy, music therapy, physiotherapy and support of family care takers by social service. This specialised inpatient palliative care intervention was offered to all of the sarcoma patients.

Symptom burden was measured with a standardised palliative base assessment questionnaire on admission and at discharge. It consisted of an evaluation of the Eastern Cooperative Oncology Group performance status (ECOG), ranking from 0 to 5, which was first published by Oken et al. in 1982 [16], pain (numeric rating scale, NRS), a German version of the National Comprehensive Cancer Network (NCCN) distress thermometer (a self-reported tool for cancer patients to screen for symptoms of distress using a scale from 0 to 10) [17], MIDOS (minimal documentation system, the German version of the Edmonton Symptom Assessment Scale) and personal situational challenges. MIDOS corresponds to a self-assessment of patients in palliative care indicating e.g. the intensity of drowsiness, nausea, constipation, dyspnea, weakness, fatigue, anxiety, lymphedema and well-being on different scales. MIDOS ranges from 0 to 3, MIDOS 0 representing the absence of the symptom, MIDOS 3 indicating a severe manifestation. It was developed to facilitate and to standardise the evaluation of symptom clusters of cancer patients. Validation of the MIDOS was first published in 2000 by Radbruch et al. [18]. The MIDOS symptom score summarises the symptom burden and consists of a summation of each MIDOS score for all symptoms (no symptom = 0 points/MIDOS 0, severe symptom = 3 points/MIDOS 3; maximum 48 for 16 symptoms in total).

Additional information on pain medication was collected from patient records.

Data analysis was performed using IBM SPSS Statistics 25 (IBM Corp., Armonk, N.Y., USA) and Microsoft Excel 14.4.7. The following nonparametric tests were used: Wilcoxon signed ranks test for metric parameters as well as McNemar test for dichotomic parameters. All *p* values were two-sided, and *p* < 0.05 was considered statistically significant. Concerning the duration of hospitalisation, generalized estimating equation (GEE) was combined with paired t tests and Sidak correction.

## Results

### Patient cohort and interventions

In our retrospective analysis 53 palliative care interventions in 34 STS patients were analysed. Thirty-three percent of the patients (n_p_ = 11) received more than one palliative care intervention (21%, n_p_ = 7 more than three). In our cohort, the median disease duration before admission to the palliative care unit was 24 months (range 1–125 months).

85% of patients (n_p_ = 29) had metastases at admission, 15% had locally advanced disease (n_p_ = 5). In contrast, only 59% of patients (n_p_ = 20) were in a palliative treatment situation at the time of diagnosis (metastasised or locally advanced). The majority of patients (94%, n_p_ = 32) had already received one or more regimen of chemotherapy, and 74% of patients (n_p_ = 25) had received radiation before their first intervention. The same number of patients (74%) had received surgery. The median patients’ age was 59 years (y) (range 21-80y). Please see Table 1 for detailed patients’ characteristics.

The majority of admissions was from home (70%, n_i_ = 37). The mean duration of hospitalisation was 15 days (d) (range 2-36d). Analysing the different subgroups (one, two and three or more interventions) there was a significant difference concerning the duration of hospitalisation between the first (mean 17d) and the second intervention (mean 11d, *p* = 0.007) as well as between the second and the third intervention (mean 18d, *p* = 0.047). Patients who died on the ward had comparable durations of inpatient interventions (13d, range 2–32).

Since parts of the palliative base assessment questionnaires were filled out by the patients themselves, on admission only 47% (n_i_ = 25) were filled out completely (excluding BMI), 45% (n_i_ = 24) were incomplete, and 8% (n_i_ = 4) were not filled out. These numbers decreased further at discharge: nearly the same number of questionnaires were filled out completely (45%, n_i_ = 18), but only 30% (n_i_ = 12) were filled out partly, and 25% (n_i_ = 10) were not filled out at all. Comparing the numbers on admission, patients who died on the ward only completed the form in 15% (n_i_ = 2) of cases (77% (n_i_ = 10) incomplete, 8% (n_i_ = 1) not filled out). The most frequent missing feature was the distress thermometer. Therefore, for subsequent analysis, only a reduced number of interventions was included, respectively: pain n_i_ = 53, ECOG n_i_ = 34, MIDOS n_i_ = 28, BMI n_i_ = 5, distress n_i_ = 13.

### Palliative care intervention

The predominant indication for admission to the palliative care unit was pain (n_i_ = 23, 67% of patients), followed by weakness (n_i_ = 15, 28%) and symptomatic tumor progression (n_i_ = 15, 28%).

#### Pain management

**Fig. 1:**
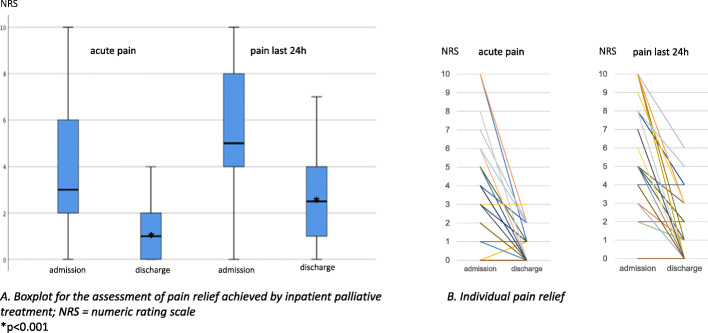
Pain relief. **a** Boxplot for the assessment of pain relief achieved by inpatient palliative treatment; NRS = numeric rating scale, *p < 0.001. **b** Individual pain relief

Regarding pain medication, 68% (n_i_ = 36) of patients were already treated with opioid medication before admission, the majority of them at a high WHO cancer pain ladder level (9% (n_i_ = 5) WHO level II, 59% (n_i_ = 31) WHO level III). 23% (n_i_ = 12) of patients were admitted without any pain medication. Palliative care intervention led to a significant reduction of pain: median NRS for acute pain was reduced from 3 to 1 (*p* < 0.001), while the pain experienced within the last 24 h before admission and at discharge was reduced from 5 to 2 (p < 0.001, see Fig. 1). 32% (n_i_ = 17) of patients received an intensification of medication according to the WHO cancer pain ladder. In contrast, patients who already received potent opioids (WHO III) stood to benefit from an opioid rotation (35%, n_i_ = 11/31) and/or a change in the route of administration (39%, n_i_ = 12), e.g. transdermal to intravenous. Notably, in 28% (n_i_ = 15) pain medication was not changed (see Table 2). Patients who died throughout hospitalisation had higher pain levels on admission (median NRS acute pain: 4, median 24 h: 6). Due to the clinical deterioration throughout the hospital stay in these patients, no reliable data could be documented on the development of individual pain perception.

**Table 2:** Drug usage for pain relief

	Total (*n* = 53)	%
**Medication on admission**		
WHO 0	12	23
WHO I	5	9
WHO II	5	9
WHO III	31	59
**Medication at discharge**		
No change	15	28
Step up	17	32
Opioid rotation	11	21
Change in administration	14	26
reduction	4	8
**WHO III on admission**		
opioid rotation at discharge	11 of 31	35
Change in administration at discharge	12 of 31	39

*n* number

#### Performance status

Interestingly, the median ECOG did not change throughout the palliative care intervention (median of 3 on admission and at discharge).

#### Stress level

**Fig. 2:**
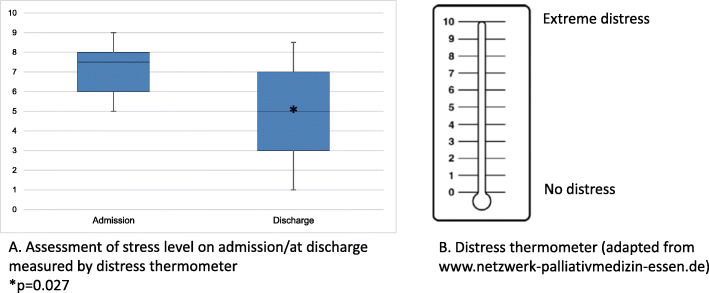
Stress level. **a** Assessment of stress level on admission/discharge measured by distress thermometer; *p = 0.027. **b** Distress thermometer (adapted from www.netzwerk-palliativmedizin-essen.de)

The median psychosocial stress level, according to the distress thermometer, was significantly reduced from 7.5 to 5 (*p* = 0.027) (see Fig. 2).

#### Symptom burden

**Fig. 3:**
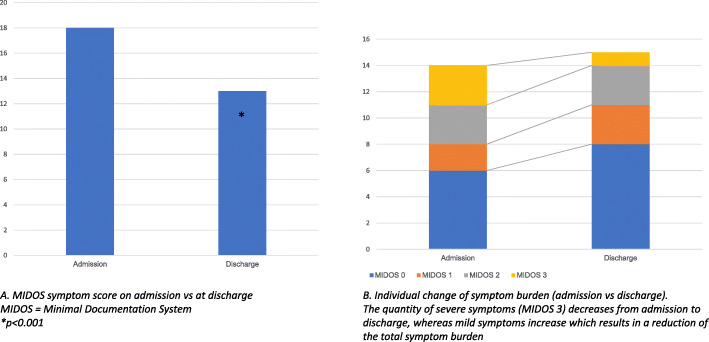
*MIDOS symptom burden.* **a** *MIDOS symptom score on admission* vs. at discharge; MIDOS = Minimal documentation system, *p < 0.001. **b** *Individual change of symptom burden (admission* vs. *discharge). The quantity of severe symptoms (MIDOS 3) decreases from admission to discharge, whereas mild symptoms increase which results in a reduction of the total symptom burden*

Median MIDOS symptom score was reduced from 18 to 13 throughout the specialised palliative care intervention (*p* < 0.001, see Fig. 3). Regarding the predominant severe symptoms (MIDOS 3) weakness and fatigue, patients benefited from the palliative care intervention. In particular, this consisted of specific spatial conditions, e.g. single-bed-room, shared kitchen, terrace, and the multiprofessional team approach at the palliative care unit. The available supportive programs included psychooncologic counselling (as support for the whole family) as well as depending on the respective symptom music therapy (e.g. for pain, distress), physiotherapy (e.g. for lymphedema, fatigue) in addition to optimisation of general care and medication (antiemetics, laxatives).

While 57% (n_i_ = 16) reported weakness on admission, the rate dropped to 29% (n_i_ = 8) at discharge (*p* = 0.008). In concordance, fatigue dropped from 43% (n_i_ = 12) on admission to 18% (n_i_ = 5) at discharge (*p* = 0.004). Other symptoms such as dyspnea, nausea and constipation were only present in less than 20% (dyspnea: 18%, nausea: 7%, constipation: 7%). For details on different symptoms please refer to Table 3.

**Table 3:** only including symptoms classified as severe (= MIDOS 3) by patients

	*n* = 28	
Symptom	Admission	Discharge
Weakness	16 (57%)	8 (29%)
Fatigue	12 (43%)	5 (18%)
Dyspnea	5 (18%)	1 (4%)
Insomnia	5 (18%)	1 (4%)
Fear	5 (18%)	2 (7%)
Loss of appetite	5 (18%)	3 (11%)
Family distress	5 (18%)	2 (7%)
Need for assistance in ADL	4 (14%)	6 (21%)
Lymphedema	4 (14%)	0 (0%)
Supply problems	3 (11%)	2 (7%)
Wound lesions	3 (11%)	0 (0%)
Nausea	2 (7%)	0 (0%)
Constipation	2 (7%)	2 (7%)
Depression	2 (7%)	0 (0%)

*n* number*, ADL* activities of daily living

#### Nutritional status

The BMI on admission and at discharge was documented for only 9% of visits (n_i_ = 5). Except for one patient, there was a tendency towards an improvement of anorexia in the remaining n_i_ = 4 patients. This was achieved by enteral and/or partly parenteral high caloric nutrition as well as appetite-enhancing drugs.

#### Patient discharge management

After completing the specialised palliative care intervention, the majority of our STS patients (58%, n_i_ = 31) could be discharged home, 11% of patients (n_i_ = 6) were transferred to a hospice and 6% (n_i_ = 3) to other institutions such as geriatric clinics for further physiotherapeutic treatment. Twenty-five percent (n_i_ = 13) of our patients died at the palliative care unit due to their advanced disease. For 32% (n_i_ = 10) of the discharged patients, a continuation of antineoplastic therapy (e.g. chemotherapy or radiation) was planned, and 42% (n_i_ = 13) of the discharged patients were provided with additional supportive care by a specialised palliative home care team.

## Discussion

### Main findings/results of the study

This study of 34 STS patients admitted for specialised palliative care intervention clearly demonstrates the effectiveness of these interventions documented by a standardised palliative base assessment. In detail, our patients experienced a significant reduction of pain, an improvement of symptom burden measured by the MIDOS symptom score and a decreased stress level. Therefore, our analysis demonstrates that specialised palliative care intervention leads to significant symptom relief and is useful for patients with advanced STS.

The importance and effectiveness of palliative care interventions have been shown for multiple oncologic diseases and are increasingly accepted [19–21]. Notably, the early integration of palliative care leads to an OS benefit which was first shown by Temel et al. in advanced lung cancer [22, 23]. In a variety of advanced cancer diseases integration of palliative care has a significant impact on patient outcome, quality of care, length of hospital stay and hospital costs [20]. Additionally, the involvement of a specialised palliative care team reduces acute care hospital treatments [24] and leads to a less aggressive therapeutic approach during end-of-life care as well as to higher levels of satisfaction among patients and their relatives [23]. Consequently, the early inclusion of palliative care within the first 8 weeks after the diagnosis of advanced cancer disease is now part of national and international clinical practice guidelines [25, 26].

Palliative care in an outpatient setting focusses on coping and support, symptom control, decision-making and future planning [27]. In general, reasons for admittance to an inpatient palliative care unit are symptom management, support for distressed families, or care for the imminently dying patient [20]. There are little data directly comparing community- and hospital-based palliative care. Generally, patients requiring a hospital stay often have a higher symptom burden, or a faster disease progression limiting the necessary transitions in their homes. In the inpatient setting, a decrease of symptom burden can often be enabled faster. For instance, an optimisation of the opioid medication requires frequent clinical feedback, which cannot always be realised in the outpatient setting.

So far, only limited data are available on the specific challenges of palliative care interventions in the context of STS. Similarities can be found comparing our patients to other well-defined cohorts with different oncologic diseases receiving specialised palliative care intervention, especially when being integrated early into the therapeutic schedule [9, 23, 24, 28]. Patients with advanced STS are known to require multidisciplinary approaches due to the aggressiveness of the heterogeneous diseases and the high occurrence of a severe symptom burden [6]. Additionally, treatment of advanced and metastasised STS often includes repeated surgical interventions, which may be associated with complications and/or mutilating procedures. Furthermore, the majority of therapeutic approaches in advanced or metastatic STS comprises of chemotherapy and/or radiotherapy, possibly resulting in diverse side effects. STS often occurs at a younger age, leading to explicitly challenging therapeutic demands. Especially in younger patients, maintenance and/or recovery of abilities regarding activities of daily living (ADL) is very important. Pain, weakness and fatigue have been the predominant indications for admission in our cohort. Nevertheless, the median NRS for acute pain at admission was not very high (median 3). In our experience, patients with chronic pain often underestimate their actual pain level referring to the NRS. Accordingly, the mental burden of pain may not directly correlate with the respective pain level. Presumably, the majority of patients would retrospectively estimate their acute pain level on admission higher than they did in the situation of admission itself. Thus, the documented pain level within the previous 24 h was significantly higher in most cases (median 5).

Our data compare well to Gough et al. on patients with advanced STS, who have documented pain, fatigue and sleep disturbances as the most frequent symptoms [6]. To our knowledge, there are no published data concerning an early integration of palliative care in patients with sarcoma. Even though we aim to integrate palliative care early in the course of disease of our patients, some patients were admitted late (median disease duration until first admission 24 months, maximum 125 months), had aggressive disease or unfortunately died throughout the repeated palliative care interventions. These circumstances can partially explain why 25% of patients died before they were discharged. Another reason for this high percentage might be a less aggressive anti-tumour therapeutic approach in those patients.

Published literature covering an end-of-life setting describes symptoms like dyspnea and fever more predominantly than in our study. Our analysis also does not entirely compare to other early palliative care interventions, which are most often realised in an outpatient and/or community-based setting [9, 27, 29].

### Strength and weaknesses/limitations of the study

Our study is limited by the sample size of 34 patients and the known restrictions of single-centre evaluation. Furthermore, for many of theinterventions data was incomplete. This was in part due to theself-assessment of some outcomes by the patients, a high symptom burden and a sometimes occuring communication barrier. Information about e.g. the pain level, which is simple to assess with the NRS, was available for all interventions. In contrast, information about more complex questions such as MIDOS and the distress thermometer was less often complete. Additionally, there were obviously no follow-up data about the deceased patients at discharge. To evaluate the benefit of the intervention for these patients, follow-up assessments could have been done earlier during the stay.

Besides the cohort size of our study, the heterogeneity of sarcomas as well as the retrospective character of our analysis make universal conclusions difficult, and in accordance our data should be validated prospectively and in a larger patient cohort.

However, even though the optimal assessment for QoL in patients with STS is not yet defined, and despite our small patient population, we could show clinically relevant effects and we still consider our data as profoundly useful as published information on these patients remains sparse. Our study emphasises the importance of palliative care intervention in advanced oncologic disease, specifically in STS patients, and even gives further evidence to support the earlier integration of palliative care intervention in STS patients. Therefore, we recommend an early integration of palliative care measurement in the first 8 weeks, according to ASCO clinical practice guidelines [25].

Some of our patients showed pain relief optimised without any change in medication. Besides the amelioration achieved by causal treatment of the pain source, other essential factors are influencing the pain level. For instance, an intensified individual support structure including psychooncologic counselling, music therapy and a reduction of psychosocial stress level, in general, may also influence the pain intensity in the individual patient.

BMI was only evaluable in a tiny subset of patients (n_i_ = 5,9%). In general, the administration of parenteral nutrition might be considered in patients with a survival of more than some weeks, only [26]. Despite the small sample size, in n_i_ = 4 (8%) patients, we found a discrete gain of body weight by optimisation of antiemetic treatment and/or appetite increase as well as the provision of high caloric nutrition if indicated.

### What this study adds

To our knowledge, this is the first report in STS patients analysing hospital-based palliative care intervention, which does not focus on end-of-life care, but on palliative care intervention throughout the entire course of the disease.

## Conclusion

Our data show the impact of specialised palliative care interventions with a multiprofessional approach on symptom relief and quality of life in patients with advanced STS. Further exploration of the effects of early integration of palliative care on symptom relief, quality of life and the possible improvement of overall survival in STS patients is warranted. Analyses in larger cohorts are warranted to answer these questions.

For this purpose, patients with suspected STS should be transferred to a specialised centre led by an interdisciplinary team to complete histologic workup and initiate causal therapy. This could simultaneously enable an early standardised screening for physical and psychosocial symptoms. With repeated assessments during the course of their disease, subgroups with special need for intensified support could be identified.

## Data Availability

Data supporting the results reported in the article are available from the authors upon reasonable request and with permission of Charité–Universitätsmedizin Berlin, corporate member of Freie Universität Berlin, Humboldt-Universität zu Berlin, and Berlin Institute of Health, Department of Hematology, Oncology, and Tumor Immunology, Campus Virchow-Klinikum, Berlin, Germany.

## Abbreviations

ADL: activities of daily living
ASCO: American Society of Clinical Oncology
BMI: body mass index
d: days
ECOG: Eastern Cooperative Oncology Group
e.g.: exempli gratia, for example
GEE: generalised estimating equation
h: hours
MIDOS: Minimal Documentation System
MPNST: Malignant peripheral nerve sheath tumor
N_i_: number of interventions
n_p_: number of patients
NCCN: National Comprehensive Cancer Center
NRS: numeric rating scale
OS: overall survival
PFS: progression free survival
QoL: quality of life
PROs: patient reported outcomes
STS: soft tissue sarcoma
WHO: World Health Organisation
